# Supplementary material for: Genome-Wide Association Study of Sweet Potato Storage Root Traits Using GWASpoly, a Gene Dosage-Sensitive Model
Source: Int J Mol Sci. 2024 Oct 31;25(21):11727. doi: 10.3390/ijms252111727 (PMC11546673; doi:10.3390/ijms252111727)
Supplement: Supplementary file 1 [file ijms-25-11727-s001.zip › ijms-3244576 Supplemental Figure.pdf]

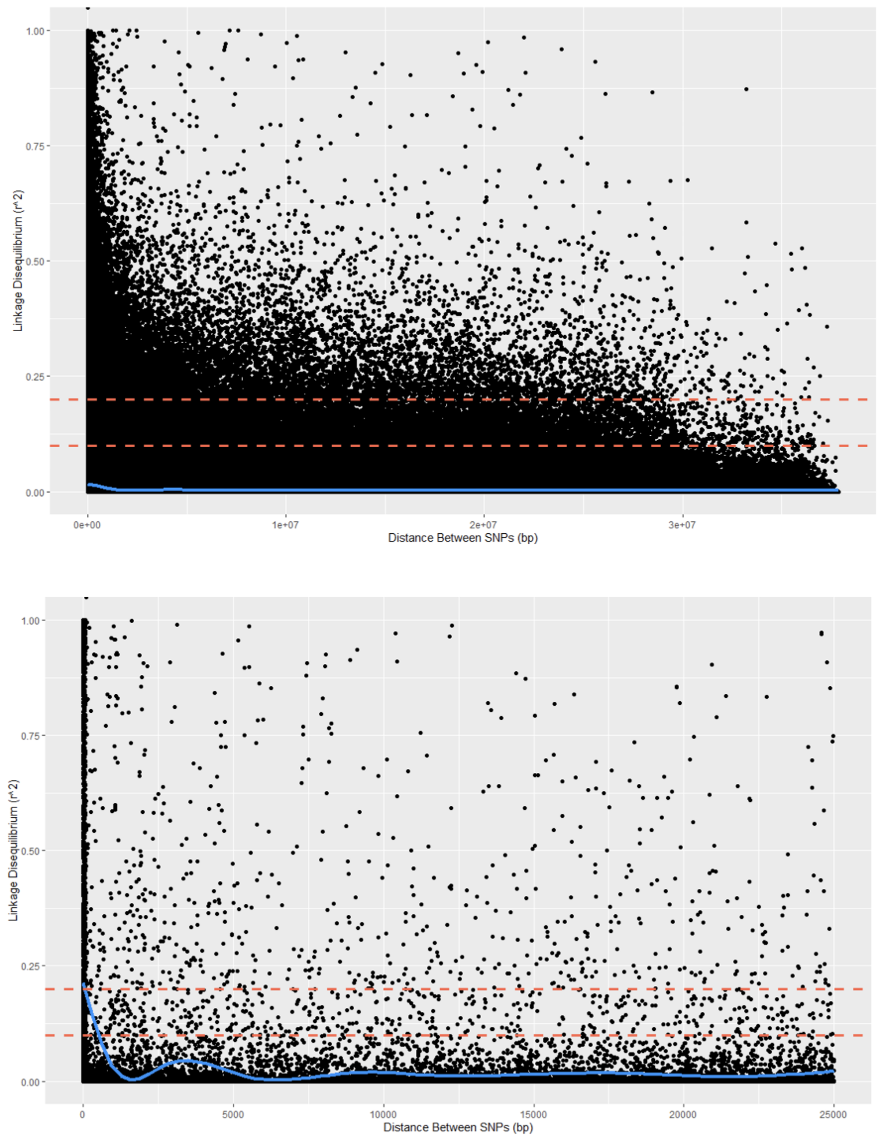

Figure S1. Linkage disequilibrium (r^2) scatterplots of SNP base pair (bp) intervals from the 384-accession breeder subset. Horizontal dashed lines at 0.1 and 0.2 (r^2) represent LD thresholds. LD decay is illustrated using a generalized additive model (gam) to fit the distribution of points (blue curve). Plots of (A) maximum bp and (B) limited 25 000 bp intervals were generated from the same dataset.

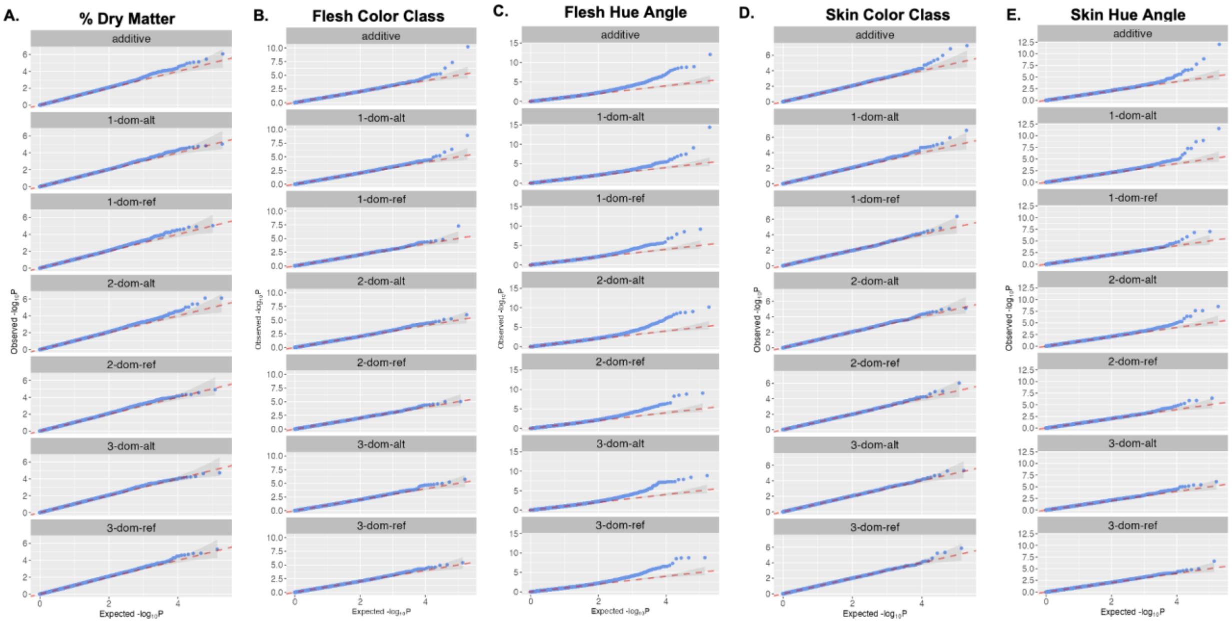

Figure S2. QQ Plots of GWAS data. For each genetic model and trait tested – (A) dry matter, (B) flesh color, (C) flesh hue angle, (D) skin color, and (E) skin hue angle – the negative $\log_{10}$ of the p-values expected by chance versus the the negative $\log_{10}$ of the observed p-values are plotted.

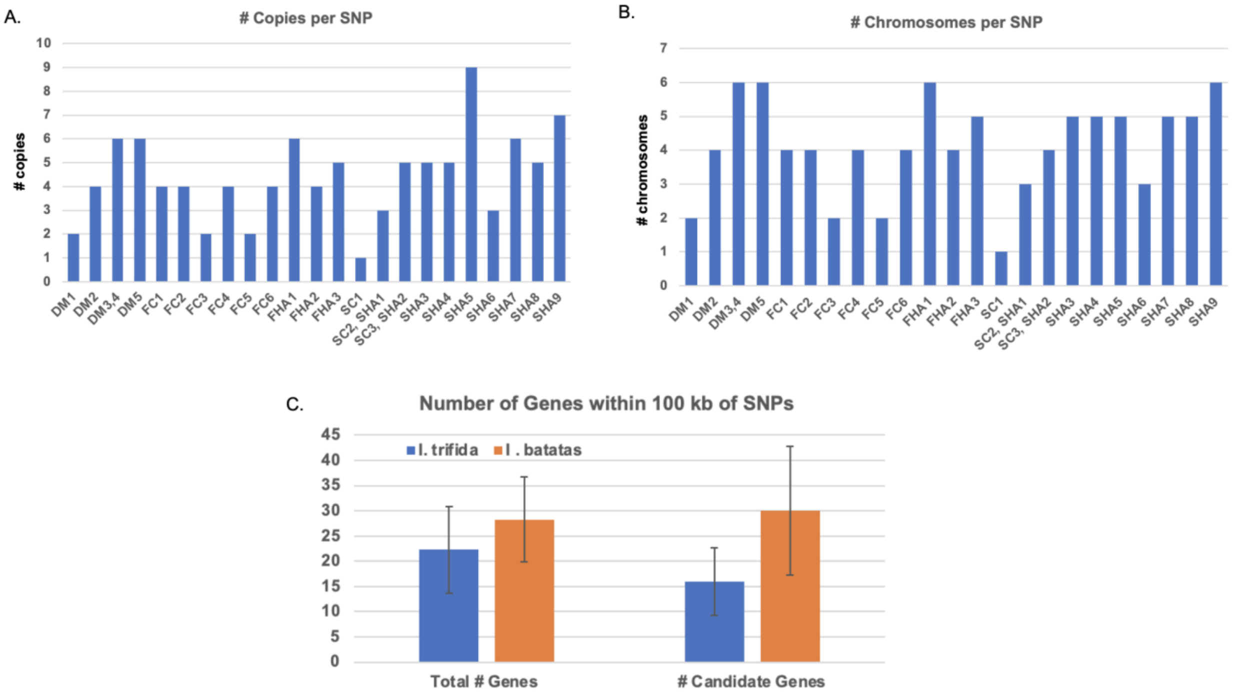

Figure S3. **(a) Number of copies of each SNP in the *I. batatas* 'Beauregard' genome, (b) number of chromosomes containing each SNP in the *I. batatas* 'Beauregard' genome, and (c) total number of genes and candidate genes identified within 100 kB of each SNP in the *I. trifida* and *I. batatas* genomes.**
